# T7 RNA polymerase catalyzed transcription of the epimerizable DNA lesion, Fapy•dG and 8-oxo-2′-deoxyguanosine

**DOI:** 10.1016/j.jbc.2024.107719

**Published:** 2024-08-29

**Authors:** Shijun Gao, Peini Hou, Dong Wang, Marc M. Greenberg

**Affiliations:** 1 Department of Chemistry, Johns Hopkins University, Baltimore, Maryland, USA; 2 Division of Pharmaceutical Sciences, Skaggs School of Pharmacy and Pharmaceutical Sciences, University of California, San Diego, La Jolla, California, USA; 3 Department of Cellular and Molecular Medicine, School of Medicine, University of California, San Diego, La Jolla, California, USA; 4 Department of Chemistry and Biochemistry, University of California, San Diego, La Jolla, California, USA

**Keywords:** DNA damage, nucleic acid chemistry, transcription, RNA polymerase, mutagenesis in vitro, enzyme kinetics

## Abstract

Fapy•dG (*N*6-(2-deoxy-α,β-D-erythro-pentofuranosyl)-2,6-diamino-4-hydroxy-5-formamidopyrimidine) and 8-OxodGuo (8-oxo-7,8-dihydro-2′-deoxyguanosine) are major products of 2′-deoxyguanosine oxidation. Fapy•dG is unusual in that it exists as a dynamic mixture of anomers. Much less is known about the effects of Fapy•dG than 8-OxodGuo on transcriptional bypass. The data presented here indicate that T7 RNA polymerase (T7 RNAP) bypass of Fapy•dG is more complex than that of 8-OxodGuo. Primer-dependent transcriptional bypass of Fapy•dG by T7 RNAP is hindered compared to 2′-deoxyguanosine. T7 RNAP incorporates cytidine opposite Fapy•dG in a miniscaffold at least 13-fold more rapidly than A, G, or U. Fitting of reaction data indicates that Fapy•dG anomers are kinetically distinguishable. Extension of a nascent transcript past Fapy•dG is weakly dependent on the nucleotide opposite the lesion. The rate constants describing extension past fast- or slow-reacting base pairs vary less than twofold as a function of the nucleotide opposite the lesion. Promoter-dependent T7 RNAP bypass of Fapy•dG and 8-OxodGuo was carried out side by side. 8-OxodGuo bypass results in >55% A opposite it. When the shuttle vector contains a Fapy•dG:dA base pair, as high as 20% point mutations and 9% single-nucleotide deletions are produced upon Fapy•dG bypass. Error-prone bypass of a Fapy•dG:dC base pair accounts for ∼9% of the transcripts. Transcriptional bypass mutation frequencies of Fapy•dG and 8-OxodGuo measured in RNA products are comparable to or greater than replication errors, suggesting that these lesions could contribute to mutations significantly through transcription.

Decreased polymerase processivity and fidelity are fundamentally important when considering the biological effects of DNA damage (1, 2, 3). The effects of damaged DNA nucleotides (lesions) on transcription are important as transcriptional mutagenesis contributes to tumorigenesis in nonproliferating and proliferating cells (4, 5, 6). 2′-Deoxyguanosine (dG) is the most readily oxidized of the native nucleotides in DNA (7). Consequently, the lesions formed from this nucleotide have been a focus of research concerning repair and replication but less so transcription. *N*6-(2-deoxy-α,β-D-erythro-pentofuranosyl)-2,6-diamino-4-hydroxy-5-formamidopyrimidine (Fapy•dG) and 8-oxo-7,8-dihydro-2′-deoxyguanosine (8-OxodGuo) (8) are derived from a common intermediate (**1**, Fig 1) and the lesions produced in the largest amounts from dG oxidation (2, 7, 9, 10, 11, 12, 13). The effects of Fapy•dG and 8-OxodGuo on DNA replication have been evaluated in the test tube and in cells (14, 15, 16, 17, 18, 19, 20, 21). Of the two lesions, 8-OxodGuo is more promutagenic than Fapy•dG in *Escherichia coli*, but the latter has exhibited greater effects on replication in mammalian cells. Transcriptional bypass of 8-OxodGuo has also been examined (22, 23, 24, 25, 26, 27, 28, 29, 30). However, the community's understanding of the consequences of encounters between RNA polymerases and Fapy•dG has lagged, and only recently have the first reports concerning the lesion's impact on RNA polymerase II (pol II) activity been published (31, 32). The effects of Fapy•dG on T7 RNA polymerase (T7 RNAP), a model RNA polymerase, are described below. For comparison, some experiments are carried out side by side on otherwise identical DNA constructs containing 8-OxodGuo.

**Figure 1:**
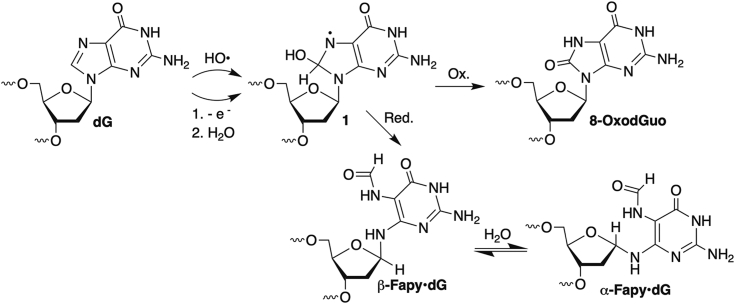
**Formation of Fapy•dG and 8-OxodGuo.** Fapy•dG, N6-(2-deoxy-α,β-D-erythro-pentofuranosyl)-2,6-diamino-4-hydroxy-5-formamidopyrimidine; 8-OxodGuo, 8-oxo-7,8-dihydro-2′-deoxyguanosine.

When 8-OxodGuo in the transcribed strand was bypassed by T7 RNAP in the test tube, the lesion had little if any impact on the readthrough frequency in two of three independent studies (22, 23). In a third, more preliminary report, 8-OxodGuo blocked T7 RNAP bypass more effectively (24). T7 RNAP preferentially inserted adenosine opposite 8-OxodGuo over cytosine, indicating that the lesion could be highly mutagenic (22). When paired with thymidine to prevent base excision repair from removing the lesion prior to transcription in *E. coli*, 8-OxodGuo also directed T7 RNAP to incorporate high levels of adenosine in transcripts (25). It is unknown whether pairing 8-OxodGuo with thymidine induced higher levels of the *syn*-conformer (Fig. 2*B*), which would encourage adenosine incorporation through presentation of the Hoogsteen face to the polymerase and incoming nucleotide triphosphate (NTP) (33).

**Figure 2:**
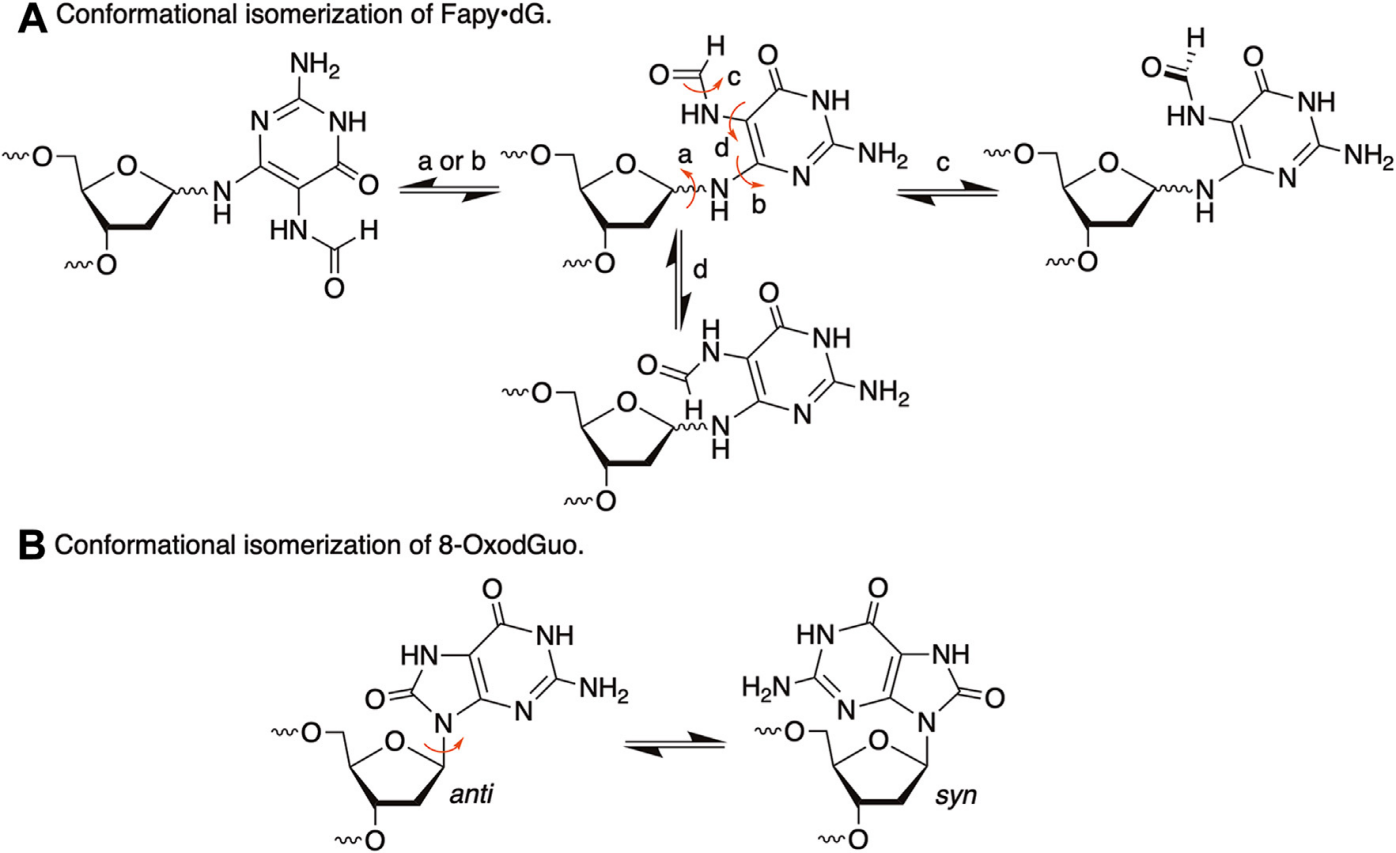
**Conformational isomerization of Fapy•dG and 8-OxodGuo.** 8-OxodGuo, 8-oxo-7,8-dihydro-2′-deoxyguanosine; Fapy•dG, N6-(2-deoxy-α,β-D-erythro-pentofuranosyl)-2,6-diamino-4-hydroxy-5-formamidopyrimidine.

Fapy•dG can adopt several stereoisomeric poses within the binding cavity of a polymerase. This plasticity is partly attributable to its unusual ability to epimerize between α- and β-configurational isomers (Fig. 1). The configuration(s) adopted by Fapy•dG depends upon the opposing nucleotide and is dynamic even within the crystalline state (34). Cleavage of the imidazole ring within the guanine from which Fapy•dG is derived provides the lesion with greater conformational flexibility than most nucleotides. For instance, rotation about the C-N bond (Fig. 2*A*; a, b) affects the orientation of the pyrimidine hydrogen bonding groups with respect to the major and minor grooves of a duplex (34). The formamide group is also free to rotate and potentially interact with incoming triphosphates or even adjacent nucleotides within the Fapy•dG-containing DNA strand (Fig 2*A*; c, d). The flexibility of Fapy•dG has been captured in cocrystal structures with DNA polymerase β and pol II, in which the lesion adapts to accommodate pairing with an incoming cytidine or adenine (31, 34). In this study, we examined the kinetics of Fapy•dG bypass in primer- and promoter-dependent transcription reactions by T7 RNAP. For comparison, the promoter-dependent experiments were carried out side by side with DNA containing 8-OxodGuo.

## Results

### Nucleic acid substrates

Experiments were carried out using miniscaffolds (**2**, **3**) or double-stranded vectors containing nucleotides of interest at defined sites. The miniscaffolds were comprised of chemically synthesized oligonucleotides. The vectors were prepared from pTGFP-T7-Hha10 plasmid as described by the Wang group (35). Oligonucleotides containing Fapy•dG were synthesized on solid-phase support as described by Yang and characterized by mass spectrometry (Fig. S8) (36).

### Primer-dependent run-off transcription by T7 RNAP is partially blocked by Fapy•dG

**Figure 3:**
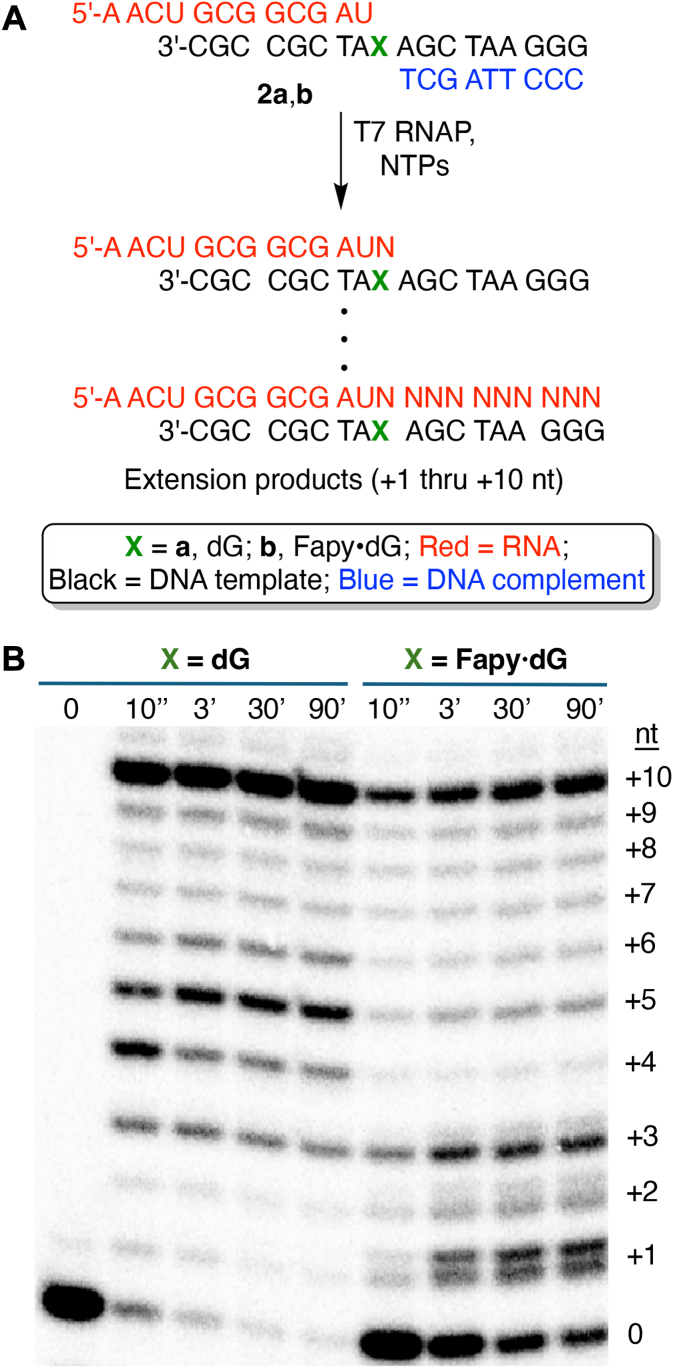
**Run-off transcription by T7 RNA polymerase using miniscaffolds.***A*, T7 RNAP (240 nM) extension of miniscaffold (20 nM) containing dG (**2a**) or Fapy•dG (**2b**) in the presence of native NTPs (1 mM each). *B*, autoradiogram showing time-dependent transcription product formation. Fapy•dG, N6-(2-deoxy-α,β-D-erythro-pentofuranosyl)-2,6-diamino-4-hydroxy-5-formamidopyrimidine; T7 RNAP, T7 RNA polymerase.

When dG is present in the template (**2a**, Fig. 3), only a small amount (6%) of starting material remained at the first time point (10 s). Approximately 49% of full-length product (+10 nt) was formed during the same time. A build-up of intermediate-length transcripts (+3 - +6 nt) was also observed for dG miniscaffold, which is potentially due to a sequence-dependent transcriptional pause. Transcripts corresponding to addition of a nucleotide opposite dG (+1 nt) and extension one nucleotide past this point (+2 nt) did not accumulate. In sharp contrast, when Fapy·dG (**2b**) is present in the template, 14% of the starting material (0 nt) remained unreacted after 90 min (Fig. 3). Furthermore, a significant amount of product(s) resulting from translesion incorporation built-up over time (+1 nt, 23% at 90 min). Product(s) resulting from an additional nucleotide (+2 nt), corresponding to single nucleotide extension past Fapy•dG in **2b**, were also observed, albeit in lower amounts. In addition, transcripts resulting from nucleotide incorporation opposite Fapy•dG (+1 nt) and extension up to two nucleotides past this point (+2, +3 nt) appear as two separate bands in the 20% denaturing gel, suggesting incorporation of different nucleotides opposite Fapy•dG, a qualitative indication of its promutagenicity. The faster migrating product(s) resulting from +1, +2, and +3 nucleotides in the Fapy•dG miniscaffold (**2b**) comigrate with the corresponding product generated upon bypass of dG (**2a**). This suggests that the faster moving bands in the 13 to 15 nt long transcripts arise from C incorporation opposite Fapy•dG, although we cannot dismiss the possibility that one or more other products resulting from incorporation of other nucleotides comigrate. To demonstrate the reproducible formation of the intermediate-length run-off transcription products from the Fapy•dG, three additional replicates were carried out (Fig. S1). The products resulting from incorporation opposite Fapy•dG (+1 nt) represented by discrete bands in the denaturing gel formed in approximately equal amounts. (The faster migrating product accounted for 43 ± 1% of the +1 nt product.) However, the extension products were increasingly enriched in the faster moving band that corresponds to the product(s) that comigrated with the transcript from dG bypass (+2 nt, 66 ± 1%; +3 nt, 75 ± 1%). This suggests that the transcript resulting from C incorporation opposite Fapy•dG is extended more readily than those yielding mutated products. Consistent with this, we observed a single band of full-length (+10 nt) run-off transcription product from Fapy•dG at the same position as the corresponding product from run-off transcription with dG (**2a**), even though the intensity is much less. Overall, the run-off transcription experiment (Fig. 3) demonstrates that Fapy•dG impedes T7 RNAP and induces the enzyme to produce promutagenic products.

### Quantitative analysis of primer-dependent T7 RNAP bypass of Fapy•dG

**Figure 4:**
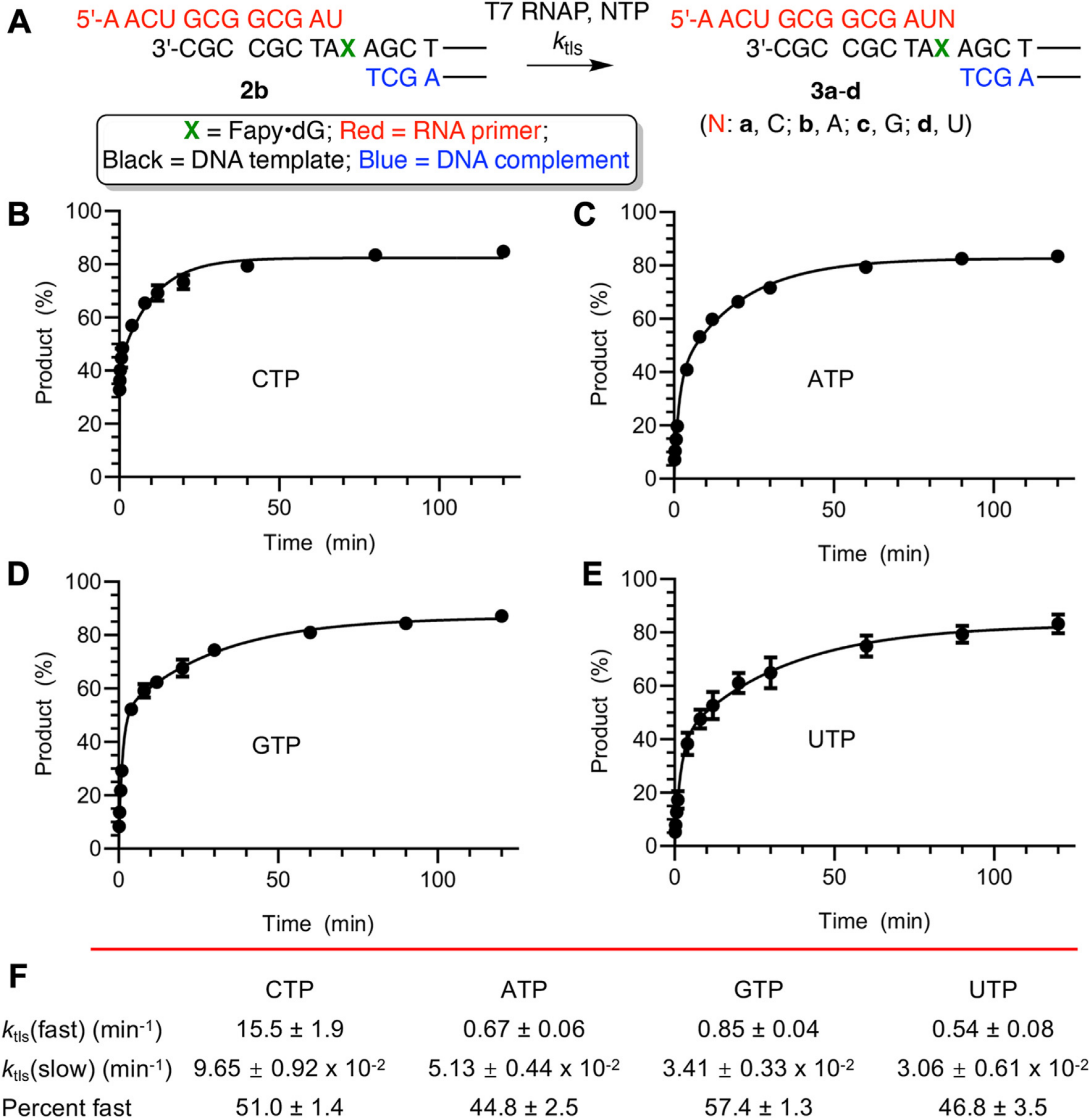
**T7 RNA polymerase nucleotide incorporation kinetics in the presence of Fapy•dG.***A*, miniscaffold substrate and products. (The complete sequence represented by the *black lines* is in Fig. 3*A*.) *B*–*E*, plots of single nucleotide incorporation opposite Fapy•dG in **2b** fitted to a double exponential. The data points on all plots are the average ± SD of three replicates. *F*, calculated values of the rate constants and percent of product accounted for by the faster of the two rate constants. Fapy•dG, N6-(2-deoxy-α,β-D-erythro-pentofuranosyl)-2,6-diamino-4-hydroxy-5-formamidopyrimidine.

**Figure 5:**
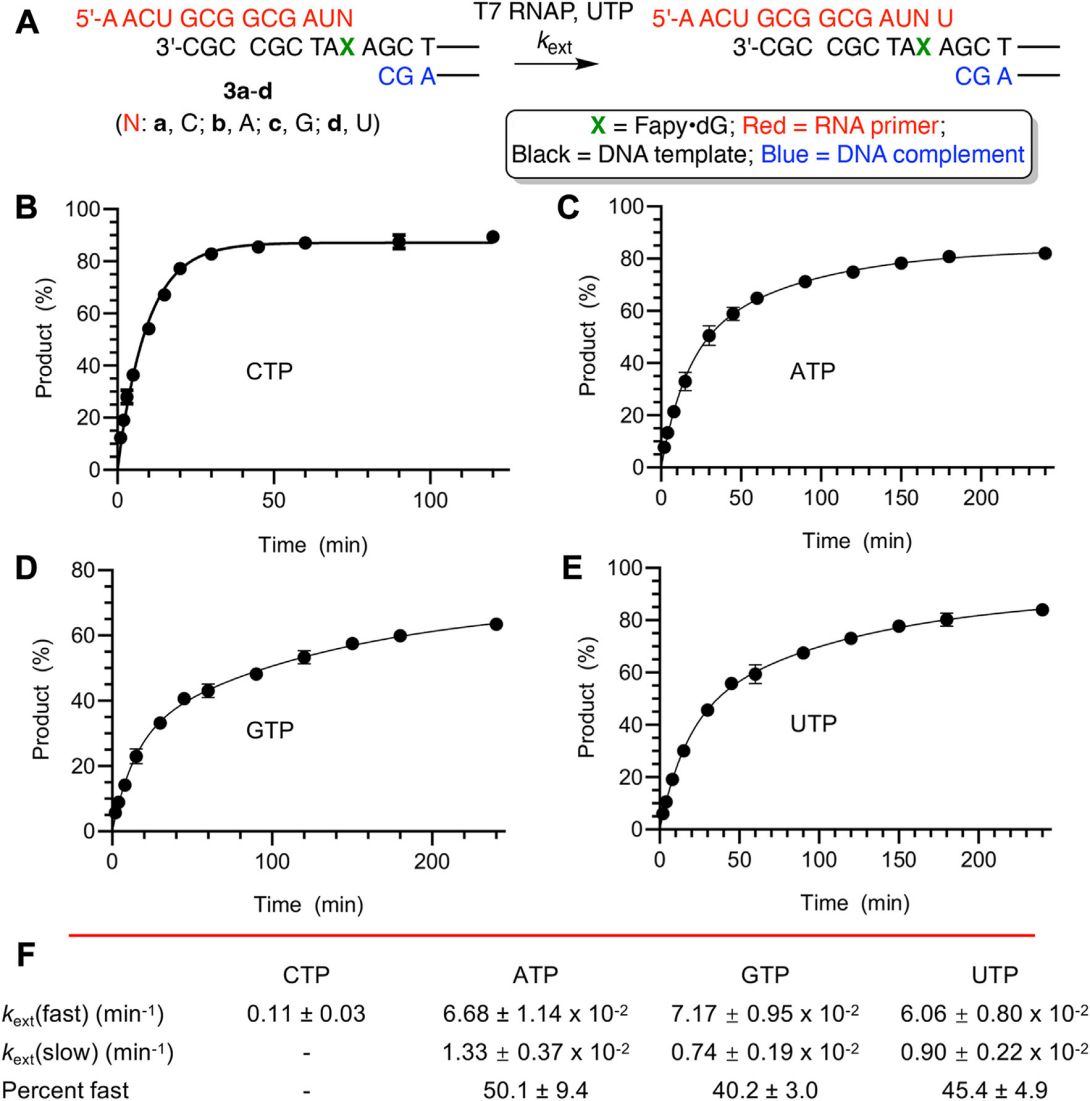
**T7 RNA polymerase nucleotide extension kinetics in the presence of Fapy•dG.***A*, miniscaffold substrate and products. (The complete sequence represented by the *black lines* is in Fig. 3*A*.) *B*–*E*, plots of single nucleotide extension as a function nucleotide opposite Fapy•dG (3a-d). The data in *B* were fitted to a single exponential. The data in *C–E* were fitted to a double exponential. The data points on all plots are the average ± SD of three replicates. *F*, calculated values of the rate constants and percent of product accounted for by the faster of the two rate constants. Fapy•dG, N6-(2-deoxy-α,β-D-erythro-pentofuranosyl)-2,6-diamino-4-hydroxy-5-formamidopyrimidine.

The impact of Fapy•dG on nucleotide incorporation kinetics was measured using miniscaffolds under single-turnover conditions (Figs 4, 5, S2 and S3). The impact of the presence of Fapy•dG configurational isomers on T7 RNAP activity was evident when measuring the kinetic rate constants for individual nucleotide incorporation opposite Fapy•dG (**2b**, Figs. 4*A* and S2). In the miniscaffold (**2b**) used to measure translesion synthesis (*k*_tls_), Fapy•dG is unpaired and thus anticipated to be present as an approximately 1:1 mixture of anomers when the DNA is bound by T7 RNAP (31, 32, 34, 37, 38). Indeed, time-dependent translesion incorporation for each nucleotide fitted a double exponential (Fig. 4, *B*–*E*) better than a single exponential (Fig. S2) for all four native NTPs. This is consistent with different *k*_tls_ for α- and β-Fapy•dG (Fig. 4*F*), as was observed when a template containing the lesion was transcribed by pol II (31). The calculated rate constants indicate that the contribution of the faster reacting isomer varies for each NTP and accounts for between 45% and 57% of the product formed (Fig. 4*F*), which is consistent with the expected ratio of α- and β-Fapy•dG present during T7 RNAP bypass. CMP incorporation opposite the faster reacting configurational isomer was already >32% complete after 5 s, and *k*_tls_(fast) (Fig. 4*F*) for this reaction (15.5 min^-1^) is likely a minimum value. The ratio of *k*_tls_(fast):*k*_tls_(slow) for CTP is very large (∼160), and based upon the known preference for β-Fapy•dG pairing with dC in duplex DNA, we propose that this isomer is the more rapid reacting one (34). It is not possible to determine which configurational isomer of Fapy•dG accounts for *k*_tls_(fast) when ATP, GTP, or UTP is present in the ternary complex. However, *k*_tls_(fast) for each of these nucleotides is between 18 and 29 times slower than the corresponding rate constant for CTP (Fig. 4*F*). The ratio of *k*_tls_(fast):*k*_tls_(slow) for ATP, GTP, or UTP is also much smaller (13, 14, 15, 16, 17, 18, 19, 20, 21, 22, 23, 24, 25) than for CTP.

Extension of a nascent primer past Fapy•dG base paired with one of the four native nucleotides (*k*_ext_) is fastest (0.11 ± 0.03 min^-1^) when C is opposite Fapy•dG (**3a**, Fig. 5 and S3). This is more than 150 times slower than the fastest *k*_tls_ measured (Fig. 4) and is consistent with observations made during run-off transcription in which the translesion synthesis product accumulates, indicating that extension is slower than incorporation (Fig. 3). UMP incorporation is well fitted by a single exponential when C is opposite Fapy•dG (**3a**, Fig. 5, *B* and *F*). This is consistent with predominant presence of a single configuration of Fapy•dG when paired with C, presumably the β-anomer, as is observed in duplex DNA (34). In contrast, addition of UMP to **3b**-**3d** is better fitted by a double exponential indicative of a mixture of α- and β-Fapy•dG opposite A, G, or U in these complexes (Figs 5, *C*–*F* and S3). The percentage of extended product in these templates accounted for by the faster reacting configurational isomer is estimated to range from 40% to 50%. The ratio of *k*_ext_ for CTP to *k*_ext_(fast) for any of the other three native NTPs is <2 (Fig. 5*F*), indicating that extension is less selective than translesion synthesis (*k*_tls_, Fig. 4*F*). Similarly, the ratio of *k*_ext_(fast):*k*_ext_(slow) for U incorporation in **3b**-**3d** varies from 5 to less than 10, which is a smaller range than for translesion synthesis (*k*_tls_(fast):*k*_tls_(slow)). Overall, the kinetic data suggest that whether transcriptional bypass will be error free or promutagenic is largely determined by the translesion synthesis step.

### Promoter-dependent T7 RNAP bypass of Fapy•dG

The competitive transcription and adduct bypass assay developed by Wang was the primary source of data on promoter-dependent transcriptional bypass (Fig. S4) (35). This double-stranded shuttle vector method provided the relative bypass efficiency compared to a control containing a dG:dC base pair that is three base pairs longer. Following RT–PCR of the transcription product, the DNA was cut with a restriction enzyme (to provide a shorter DNA to analyze), 5′-^32^P-labeled, and the products were separated by denaturing PAGE. The transcriptional bypass efficiency was determined by quantifying the four possible products containing dA, dC, dG, or T (13 nt) and deletions (12 nt) relative to the amount of product attributable to bypass of the control (16 nt). In addition, a recently reported modified version of Essigmann's restriction endonuclease and postlabeling (REAP) assay was employed to quantify individual levels of promutagenic nucleotides incorporated opposite the lesions (Fig. S5) (32, 39). Briefly, the nucleotide incorporated opposite the lesion was ultimately identified following thin layer chromatography of the corresponding 5′-^32^P-monophosphates. The radiolabeled monophosphates were obtained from the RT–PCR product via a series of enzyme transformations and denaturing PAGE separation. For comparison, bypass of Fapy•dG and 8-OxodGuo opposite the same nucleotides was studied side by side. In addition, shuttle vectors containing dA or dC opposite the lesions were utilized, because replication experiments in mammalian cells indicated these nucleotides are the ones most likely to be encountered opposite Fapy•dG and 8-OxodGuo by RNA polymerase (16, 19, 20, 40).

**Figure 6:**
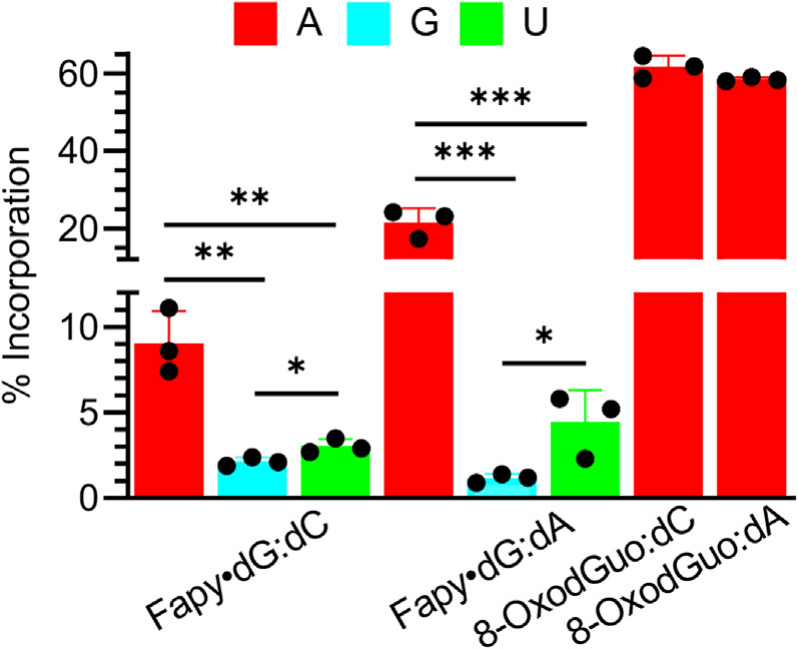
**Nucleotide misincorporation opposite Fapy•dG or 8-OxodGuo measured using the REAP assay.** Values are the average ± SD of three replicates. ∗*p* < 0.05, ∗∗*p* < 0.01, and ∗∗∗*p* < 0.001. Fapy•dG, N6-(2-deoxy-α,β-D-erythro-pentofuranosyl)-2,6-diamino-4-hydroxy-5-formamidopyrimidine; 8-OxodGuo, 8-oxo-7,8-dihydro-2′-deoxyguanosine; REAP, restriction endonuclease and postlabeling.

Fapy•dG and 8-OxodGuo bypass efficiency by T7 RNAP was greater when either lesion was paired with dC than dA (Table 1, Fig. S6). The polymerase bypassed Fapy•dG:dC even more efficiently than a dG:dC containing control. This was also observed during pol II promoter-dependent bypass of Fapy•dG:dC (32). In contrast, 8-OxodGuo bypass was moderately hindered when paired with either nucleotide, as was Fapy•dG paired with dA. The point mutation levels were considerably greater when T7 RNAP bypassed 8-OxodGuo compared to Fapy•dG. More than 55% mutations from 8-OxodGuo were detected using the competitive transcription and adduct bypass (Table 1, Fig. S6) or REAP (Figs. 6 and S7) assays. In addition, the point mutation level observed upon 8-OxodGuo bypass was the same whether the lesion was paired with dC or dA (Table 1, Fig. 6), despite the assumption that the lesion presents its Hoogsteen face within the shuttle vector to an incoming NTP when opposite the latter due to its presence in the *syn*-conformation (Fig. 2*B*) (33). In addition, adenosine was the only promutagenic nucleotide detected opposite 8-OxodGuo (Fig. 6). The outcome of Fapy•dG transcriptional bypass was more varied. The increased impediment to bypass when the lesion was paired with dA resulted in a doubling of the point mutation level as well as the generation of single-nucleotide deletions (Table 1, Fig. 6). Although adenosine was the nucleotide most frequently misincorporated, T7 RNAP also incorporated lower levels of guanosine and uridine opposite Fapy•dG.

**Table 1:** Promoter-dependent T7 RNA polymerase transcription^a^

Duplex	% Bypass^b^	% point mutation	% deletion
Fapy•dG:dC	149.7 ± 4.4	9.1 ± 0.5	n.d.
Fapy•dG:dA	57.4 ± 2.7	20.5 ± 0.1	9.2 ± 0.7
8-OxodGuo:dC	71.9 ± 5.0	55.1 ± 1.3	n.d.
8-OxodGuo:dA	57.5 ± 2.4	55.2 ± 0.1	n.d.

n.d., not detected.

a Data are the average ± SD of three replicates.

b Determined by comparing the amount of product from the lesion-containing construct to that from a control containing a dG:dC base pair.

## Discussion

Primer- and promoter-dependent transcription of Fapy•dG-containing templates by T7 RNAP bypass was carried out. Primer extension experiments indicate that the lesion moderately blocks the enzyme. A build-up of intermediate-length products corresponding to nucleotide incorporation opposite the lesion, followed by one to two nucleotide extension during run-off transcription, suggests that extension past the lesion is slower than translesion incorporation. This scenario is supported by kinetic experiments in which for even error-free nucleotide incorporation *k*_tls_ >> *k*_ext_. In the run-off transcription experiment (Fig. 3), Fapy•dG is unpaired and therefore present as an approximately equal mixture of anomers (34). One cannot discern from this experiment whether α- and β-Fapy•dG are bypassed at different rates. The run-off transcription experiment indicated that at least one nucleotide other than C is incorporated opposite Fapy•dG and that this promutagenic product is extended more slowly. Overall, the run-off transcription experiment suggested that bypass is more efficient following C incorporation opposite Fapy•dG than when T7 RNAP introduces other nucleotides opposite the lesion.

That the configurational isomers are bypassed at different rates was supported by fitting the single nucleotide incorporation kinetic data to a double exponential. The selectivity for nucleotide incorporation opposite Fapy•dG is similar qualitatively to observations in similar experiments involving pol II, which is structurally distinct from T7 RNAP (31, 41, 42). The faster reacting configurational isomer shows a large preference for C incorporation. However, T7 RNAP barely differentiates between A, G, or U misincorporation opposite either Fapy•dG configurational isomer, and the difference between *k*_tls_(fast) and *k*_tls_(slow) is much smaller than for C. Furthermore, the rate constant for C incorporation opposite the slower reacting Fapy•dG anomer is also very similar to those for the other three native nucleotides. We cannot draw any conclusions regarding which configurational isomer of Fapy•dG is responsible for *k*_fast_ nor does the same isomer have to account for the fast incorporation of each nucleotide. A significant difference between T7 RNAP and pol II is observed upon extension past the lesion (31). The rate constant for pol II extension past Fapy•dG paired with a native nucleotide in a template (*k*_ext_) is comparable to *k*_tls_. In addition to being significantly slower than *k*_tls_, *k*_ext_ for T7 RNAP varies less than twofold when the nucleotide opposite Fapy•dG is varied. This too is a difference between T7 RNAP and pol II in which extension past Fapy•dG (*k*_ext_) by the latter depends more strongly on the nucleotide incorporated opposite the lesion (31). Another major difference between T7 RNAP and pol II is that the latter has additional proofreading activities via intrinsic or TFIIS-stimulated cleavage, which preferentially cleave error-prone transcripts over error-free transcripts (31). Taken together, unlike pol II, the major fidelity checkpoint for T7 RNAP is the translesion insertion step opposite Fapy•dG.

The impact of Fapy•dG on T7 RNAP primer-dependent bypass efficiency is not readily extrapolatable to a promoter-dependent reaction when the lesion is paired with dC. This may be attributable to the predominant presence of β-Fapy•dG opposite C, which is bypassed more readily than other base pairs. In this regard, it is notable that the promoter-dependent bypass efficiency of a Fapy•dG:dA base pair in which a significant amount of the lesion is expected to be present in the α-configuration is approximately one-half that of a dG:dC base pair (34). Although other than when the nucleotide opposite Fapy•dG is (d)C, we cannot discern which configurational isomer reacts faster, we propose that the presence of two Fapy•dG anomers in primer- and promoter-dependent transcription experiments is a significant contributor to T7 RNAP blockage.

Although Fapy•dG and 8-OxodGuo were bypassed by T7 RNAP with comparable efficiency in promoter-dependent reactions, the latter yielded significantly higher levels of point mutations. The observation regarding point mutations is very different than what was observed for pol II bypass of these DNA lesions (32). In experiments with pol II, the levels of point mutations upon Fapy•dG transcriptional bypass were comparable to or greater than for 8-OxodGuo. The greater mutation frequency upon Fapy•dG bypass was accentuated for pol II when dA was paired with the lesions. Although the point mutation levels resulting from 8-OxodGuo bypass were similar to those previously reported for T7 RNAP transcription (22, 25), it is surprising that the level is the same whether 8-OxodGuo is paired with dC or dA in the shuttle vector. When paired with dA, 8-OxodGuo is expected to be present in the *syn*-conformation and present its Hoogsteen face to the incoming NTP (Fig. 2*B*), which is conducive to incorporating A (33). The resulting base pair is readily accommodated by the RNA polymerase (43). Is it possible that 8-OxodGuo readily adopts the *syn*-conformation upon T7 RNAP binding even when the lesion is paired with dC? Unfortunately, our data do not enable us to speculate as to whether this is the case.

The strong preference for misinsertion of A opposite 8-OxodGuo over G or U is consistent with the high preference for G → T transversions over other mutations during replicative bypass of this lesion (14, 15, 19, 44). Likewise, the greater variety of promutagenic bypass events of Fapy•dG by T7 RNAP during promoter-dependent transcription, in which A, G, or U is misinserted is also consistent with replication experiments (20). It is notable that replication experiments were carried out using single-stranded vectors in which α- and β-Fapy•dG are likely present in approximately equal amounts. We propose that the overall increase in point mutations observed during transcriptional bypass by T7 RNAP when Fapy•dG is paired with dA compared to dC correlates with greater amounts of the α-anomer in this template. A similar trend was observed when pol II bypasses Fapy•dG (32). Primer-dependent T7 RNAP transcription indicates that β-Fapy•dG strongly favors C translesion incorporation. However, the rate constant for C incorporation opposite the slower reacting α-anomer is comparable to those for inserting A, G, or U. In addition, in primer-dependent reactions, T7 RNAP does not strongly favor extending a primer containing any of the four native nucleotides opposite Fapy•dG over another. Hence, the presence of greater amounts of α-Fapy•dG is expected to yield higher levels of mutations, which are observed when the lesion is paired with dA. Although there is no structural precedent, it is also possible that the higher level of α-Fapy•dG present when the lesion is paired with dA gives rise to the deletions formed upon promoter-dependent T7 RNAP bypass. Given that dA incorporation is the mutation most frequently detected upon Fapy•dG replication, the increased transcriptional mutagenesis when the lesion is paired with dA inferred from these experiments could be biologically relevant.

## Experimental Procedures

### General materials and methods

Oligodeoxynucleotides made exclusively of native nucleotides were sourced from Integrated DNA Technologies (Coralville, IA). The Fapy•dG-containing oligonucleotide was synthesized following established protocols and characterized using a Bruker AutoFlex III MALDI-TOF/TOF mass spectrometer (36). Gel electrophoresis and REAP experiments on ^32^P-labeled materials were quantified using a Storm 840 phosphorimager and analyzed using ImageQuant TL software. Fapy•dG and 8-OxodGuo containing pTGFP-T7-Hha10 vectors were prepared as previously described (32, 35). [γ-^32^P] ATP was purchased from PerkinElmer (Shelton, CT). T4 polynucleotide kinase was acquired from New England Biolabs (Beverly, MA). Individual rNTPs were obtained from Promega (Madison, WI). Oligonucleotide synthesis reagents, including 8-OxodGuo phosphoramidite, were obtained from Glen Research. All other necessary materials were purchased from New England Biolabs or Sigma–Aldrich, unless stated otherwise.

### Purification of T7 RNAP

The purification of T7 RNAP was performed as previously described (45). Briefly, wild-type full-length T7 RNAP was cloned into a modified pET28a vector, which harbors 6× Histidine tag at the N terminus, followed by a tobacco etch virus protease cleavage site. For the expression of T7 RNAP, the plasmid was transformed into Rosetta 2 (DE3)-competent cells, and T7 expression was induced by 0.5 mM IPTG when OD_600_ reached 0.6, followed by overnight expression at 20 °C. Cells were harvested and resuspended in lysis buffer (20 mM Tris–HCl, pH 7.5, 200 mM NaCl, 5% glycerol, and 2 mM β-mercaptoethanol), then lysed with a microfluidizer. Pellets were removed after centrifugation, and the supernatant was incubated with Ni–NTA agarose (Qiagen) for 1 h. After incubation, the supernatant was removed and Ni–NTA column was washed with buffer A (20 mM Tris–HCl, pH 7.5, 200 mM NaCl, 5% glycerol, 2 mM β-mercaptoethanol, and 20 mM imidazole) and buffer B (20 mM Tris–HCl, pH 7.5, 200 mM NaCl, 5% glycerol, 2 mM β-mercaptoethanol, and 30 mM imidazole). To remove the 6× His tag, on-column tobacco etch virus cleavage was conducted overnight at 4 °C. After cleavage, the eluted protein was further purified with a Heparin HP column (Cytiva). The purified T7 RNPA was concentrated, fast-frozen with liquid nitrogen, and stored at −80 °C.

### T7 RNAP-mediated run-off transcription

The miniscaffold (**2a** or **2b**) was formed by heating a 5′-^32^P-labeled RNA primer (200 nM), template DNA (600 nM), and nontemplate strand DNA (800 nM) in transcription elongation buffer (20 mM Tris–HCl, pH 7.5, 40 mM KCl, 5 mM MgCl_2_, and 5 mM DTT) at 60 °C for 5 min, followed by gradual cooling to room temperature in a heat block. The miniscaffold was mixed with T7 RNAP in 1× transcription elongation buffer and preincubated at 30 °C for 30 min. The reaction was initiated by adding an equal volume of rNTP solution (2 mM each) in 1× transcription elongation buffer. The final concentration of each component was 20 nM miniscaffold, 240 nM T7 RNAP, and 1 mM rNTP (total volume 15 μl). At specific time points (10 s, 3 min, 30 min, 90 min), an aliquot (2.5 μl) was removed and mixed with formamide loading buffer (10 μl, 90% formamide, 50 mM EDTA, 0.05% xylene cyanol, and 0.05% bromophenol blue). The samples were cooled on ice and denatured at 95 °C for 5 min. Subsequently, denatured samples were analyzed by phosphorimaging following separation by 20% denaturing PAGE.

### T7 RNAP-mediated translesion transcription kinetics

Miniscaffold **2b**, prepared as described above, was mixed with T7 RNAP in 1× transcription elongation buffer and preincubated at 30 °C for 30 min. The reaction was initiated by adding an equal volume of individual rNTP solution (2 mM) in transcription elongation buffer. The final concentration of each component was 20 nM miniscaffold, 240 nM T7 RNAP, and 1 mM rNTP (total volume 30 μl). An aliquot (2 μl) of the reaction was mixed with formamide loading buffer (10 μl) at each time point and placed on ice. Quenched aliquots were denatured at 95 °C (5 min) and separated by 20% denaturing PAGE. Time points for CTP incorporation were 5 s, 10 s, 20 s, 40 s, 1 min, 4 min, 8 min, 12 min, 20 min, 40 min, 80 min, and 120 min. Time points for ATP/GTP/UTP incorporation were 10 s, 20 s, 40 s, 1 min, 4 min, 8 min, 12 min, 20 min, 30 min, 60 min, 90 min, and 120 min. Kinetic parameters were determined by fitting the data using Prism to either a single or double exponential model, represented by the following equations:

Single exponential model:

Y=Plateau×[1−exp(−kt)]

Double exponential model:

$$Y=\text{Plateau}\times \left(\frac{\text{PercentFast}}{100}\right)\times \left[1-\exp\left(-k_{1}t\right)\right]+\text{Plateau}\times \left[1-\left(\frac{\text{PercentFast}}{100}\right)\right]\times \left[1-\exp\left(-k_{2}t\right)\right]$$

Where:

• t is the reaction time (min);
• Y is the percent product;
• Plateau is the percent product at infinite time (%);
• PercentFast is the percent of the span (from Y = 0 to plateau) accounted for by the faster of the two components;
• k corresponds to the rate constant (min^-1^) for single exponential fitting;
• k_1_ and k_2_ correspond to the rate constants (min^−1^) of the fast and slow reactions, respectively, for double exponential fitting.

### T7 RNAP-mediated extension kinetics of C/A/G/U opposite Fapy•dG

The miniscaffold (**3a**-**d**), prepared as described above, was mixed with T7 RNAP in 1× elongation buffer and preincubated at 30 °C for 30 min. The reaction was initiated by adding an equal volume of 4 mM UTP solution in 1× transcription elongation buffer. The final concentration of each component was 20 nM miniscaffold, 480 nM T7 RNAP, and 2 mM UTP (total volume 30 μl). An aliquot (2 μl) of the reaction was mixed with formamide loading buffer (10 μl) at each time point and placed on ice. Quenched aliquots were denatured at 95 °C and separated by 20% denaturing PAGE. Time points for extension of C opposite Fapy•dG (**3a**) were 1 min, 2 min, 3 min, 5 min, 10 min, 15 min, 20 min, 30 min, 45 min, 60 min, 90 min, and 120 min. Time points for extension of A/G/U opposite Fapy•dG (3b-d) were 2 min, 4 min, 8 min, 15 min, 30 min, 45 min, 60 min, 90 min, 120 min, 150 min, 180 min, and 240 min. Kinetic parameters were determined by fitting the data using Prism to either the single or double exponential model described above.

### Promoter-dependent in vitro transcription

Lesion-bearing or lesion-free control plasmids were prepared as previously described and premixed with the competitor vector at a molar ratio of 3:1 (lesion:competitor) (32, 35). Following this, the plasmid mixture (500 ng) was incubated with NotI (5 U) in 1× bovine serum albumin (100 μg/ml) and 1× NEB buffer 3.1 (total volume of 20 μl) at 37 °C for 1 h. The enzyme activity was terminated by heating the reaction solution at 70 °C for 20 min. The resultant linearized vector was purified using the E.Z.N.A. Cycle Pure kit (Omega Bio-Tek), following the manufacturer’s guidelines, and eluted in elution buffer (30 μl). Subsequently, the mixture of linearized vectors (50 ng) was combined with T7 RNAP (3.5 μM, 1 μl), rNTP (2.5 mM each, 4 μl), and RNase inhibitor (40 U/μl, 0.25 μl) in T7 transcription buffer (40 mM Tris–HCl, pH 7.9, 6 mM MgCl_2_, 10 mM DTT, 10 mM NaCl, 2 mM spermidine, 0.05% Tween-20, total volume 20 μl). The mixture was incubated at 37 °C for 1 h, and then the reaction was stopped by the addition of 0.2 M EDTA (2 μl) (46).

### Purification and RT–PCR amplification of mRNA transcript

The mRNA produced via in vitro transcription was purified using the Total RNA Kit (Omega Bio-Tek) and was eluted with nuclease-free water (50 μl). Subsequently, all the purified RNA (50 μl) was mixed with 10× DNase I buffer (5 μl) and DNase I (2 U/μl, 1 μl), and the reaction was incubated at 37 °C for 30 min. The mRNA was then purified according to the manufacturer’s protocol for the DNA-free DNA Removal Kit (Invitrogen). The remaining mRNA (50 μl) was treated again with DNase I (2 U/μl, 1 μl) and 10× DNase I buffer (7.5 μl), followed by purification according to the manufacturer’s instructions. The DNA-free mRNA (6.6 μl) was combined with the primer (10 μM, 0.4 μl; 5′-TCGGTGTTGCTGTGAT-3′) in a sterile RNase-free microcentrifuge tube, heated at 70 °C for 5 min, and rapidly chilled on ice. The annealed mRNA (7 μl) was then mixed with M-MLV reverse transcriptase (200 U/μl, 0.4 μl), dNTP (10 mM each, 0.5 μl), and RNase inhibitor (40 U/μl, 0.1 μl) in the presence of the commercial 1× M-MLV reaction buffer (total volume 10 μl). The reaction was incubated at 42 °C for 1.5 h, followed by 75 °C for 15 min. For PCR, the reverse transcription reaction mixture (1 μl) was combined with primers (10 μM × 1 μl each, 5′-CTAGCGGATGCATCGACTC-3′ and 5′-GGCCGCTCTCGTCGCTCTC-3′), dNTP (10 mM, 1 μl), and Phusion High-Fidelity DNA Polymerase (2 U/μl, 0.3 μl) in 1× Phusion HF buffer (total volume 50 μl). The PCR protocol comprised the following steps: step 1: 98 °C for 30 s; step 2 (repeated for 35 cycles): 98 °C for 10 s, 60 °C for 30 s, 72 °C for 15 s; and step 3: 72 °C for 5 min. Finally, a portion of the PCR (5 μl) was assessed using a 2% agarose gel, while the remainder was purified using the E.Z.N.A. Cycle Pure kit according to the manufacturer’s instructions.

### Analysis of RT–PCR product resulting from in vitro transcription

The purified PCR product (50 ng) was combined with NcoI (10 U/μl, 0.5 μl) and SAP (1 U/μl, 0.5 μl) in 1× NEB buffer 3.1 (total volume 10 μl). The mixture was incubated at 37 °C for 1 h and was then heated to 70 °C for 20 min. Following this, the entire reaction mixture was mixed with T4 PNK (10 U/μl, 0.5 μl), ATP (100 μM, 0.1 μl), and [γ-^32^P]-ATP (10 μCi/μl, 0.5 μl) in 1× NEB buffer 3.1 (total volume 15 μl). The kinase reaction was allowed to proceed at 37 °C for 1 h and then heated to 65 °C for 20 min. Subsequently, the resulting solution was combined with SfaNI (2 U/μl, 1 μl) in 1× NEB buffer (total volume 20 μl) and incubated at 37 °C for 1.5 h. The reaction was stopped by adding 2× gel loading buffer (20 μl, 45% formamide, 0.1% bromophenol blue, 0.1% xylene cyanol, and 5 mM EDTA). An aliquot (7 μl) of the sample was separated by 30% native PAGE in 0.5× TBE buffer and run at 40 W for 5 h. Following electrophoresis, the gel was wrapped and exposed to a storage phosphor screen for >12 h. Finally, the phosphor screen was scanned using a phosphorimager to quantify the 16-mer and 12/13-mer bands, enabling the calculation of bypass efficiencies and mutation frequencies.

### REAP assay

The RT–PCR product (50 ng) was digested with Cac8I (0.5 U/μl) and SAP (0.05 U/μl) in 1× NEB buffer 3.1 (total volume 10 μl) by incubating at 37 °C for 3 h. The enzymes were inactivated by heating the reaction mixture at 70 °C for 20 min. Subsequently, the reaction mixture was supplemented with ATP (1 μM), [γ-^32^P]-ATP (0.67 μCi/μl), and T4 PNK (0.33 U/μl) in 1× NEB buffer 3.1 (total volume 15 μl), and incubated at 37 °C for 1 h. T4 PNK was deactivated by heating at 65 °C for 20 min. Following this, NsiI (0.1 U/μl) was added to the mixture in 1× NEB buffer (total volume 20 μl) and incubated at 37 °C for 2 h. The reaction was stopped by adding 20 μl of formamide loading buffer. The mixture was separated by 20% denaturing PAGE run at 50 W for 4 h. The target region for the 21 nt fragment was excised, crushed, and eluted with a solution (200 μl) containing NaCl (0.2 M) and EDTA (1 mM) at 37 °C overnight with shaking. After removing the gel particles through filtration, the filtrate was desalted twice with 1 ml Sephadex G25 resin (Cytiva) and then dried under vacuum. The resulting residue was mixed with nuclease P1 (20 U/μl) in commercial 1× nuclease P1 reaction buffer (total volume 5 μl) and incubated at 37 °C overnight. Then, 0.5 μl (∼3000 cpm) of the nuclease P1 reaction was spotted onto the polyethyleneimine cellulose TLC plate (Fisher Scientific). The TLC plate was immersed in a tank containing saturated (NH_4_)_2_HPO_4_ (200 ml, pH 5.8). After the solvent front reached the top (∼18 h), the plates were air-dried and exposed to a storage phosphor screen.

## Data availability

Data are available upon request from M.M.G. (mgreenberg@jhu.edu).

## Supporting information

This article contains supporting information.

## Conflict of interest

The authors declare that they have no conflicts of interest with the contents of this article.

## References

[1] Dizdaroglu M. (2015). Oxidatively induced DNA damage and its repair in cancer. Mutat. Res. Rev. Mutagen..

[2] Cadet J., Davies K.J.A., Medeiros M.H.G., Di Mascio P., Wagner J.R. (2017). Formation and repair of oxidatively generated damage in cellular DNA. Free Rad. Biol. Med..

[3] Basu A.K., Essigmann J.M. (2022). Establishing linkages among DNA damage, mutagenesis, and genetic diseases. Chem. Res. Toxicol..

[4] Brégeon D., Doetsch P.W. (2011). Transcriptional mutagenesis: causes and involvement in tumour development. Nat. Rev. Cancer.

[5] Ezerskyte M., Paredes J.A., Malvezzi S., Burns J.A., Margison G.P., Olsson M. (2018). O6-methylguanine–induced transcriptional mutagenesis reduces p53 tumor-suppressor function. Proc. Natl. Acad. Sci. USA.

[6] Reijns M.A.M., Parry D.A., Williams T.C., Nadeu F., Hindshaw R.L., Rios Szwed D.O. (2022). Signatures of TOP1 transcription-associated mutagenesis in cancer and germline. Nature.

[7] Cadet J., Douki T., Ravanat J.-L. (2008). Oxidatively generated damage to the guanine moiety of DNA: mechanistic aspects and formation in cells. Acc. Chem. Res..

[8] Cooke M.S., Loft S., Olinski R., Evans M.D., Bialkowski K., Wagner J.R. (2010). Recommendations for standardized description of and nomenclature concerning oxidatively damaged nucleobases in DNA. Chem. Res. Toxicol..

[9] Dizdaroglu M., Kirkali G., Jaruga P. (2008). Formamidopyrimidines in DNA: mechanisms of formation, repair, and biological effects. Free Rad. Biol. Med..

[10] Pouget J.-P., Douki T., Richard M.-J., Cadet J. (2000). DNA damage induced in cells by γ and UVA radiation as measured by HPLC/GC-MS and HPLC-EC and comet assay. Chem. Res. Toxicol..

[11] Jaruga P., Speina E., Gackowski D., Tudek B., Olinski R. (2000). Endogenous oxidative DNA base modifications analysed with repair enzymes and GC/MS technique. Nucleic Acids Res..

[12] Liu D., Croteau D.L., Souza-Pinto N., Pitta M., Tian J., Wu C. (2010). Evidence that OGG1 glycosylase protects neurons against oxidative DNA damage and cell death under ischemic conditions. J. Cereb. Blood Flow Metab..

[13] Pouget J.P., Frelon S., Ravanat J.L., Testard I., Odin F., Cadet J. (2002). Formation of modified DNA bases in cells exposed either to gamma radiation or to high-LET particles. Radiat. Res..

[14] Shibutani S., Takeshita M., Grollman A.P. (1991). Insertion of specific bases during DNA synthesis past the oxidation-damaged base 8-OxodG. Nature.

[15] Tan X., Grollman A.P., Shibutani S. (1999). Comparison of the mutagenic properties of 8-Oxo-7,8-dihydro-2'-deoxyadenosine and 8-Oxo-7-8-dihydro-2'-deoxyguanosine DNA lesions in mammalian cells. Carcinogenesis.

[16] Kalam M.A., Haraguchi K., Chandani S., Loechler E.L., Moriya M., Greenberg M.M. (2006). Genetic effects of oxidative DNA damages: comparative mutagenesis of the imidazole ring-opened formamidopyrimidines (Fapy lesions) and 8-Oxo-purines in simian kidney cells. Nucleic Acids Res..

[17] Patro J.N., Wiederholt C.J., Jiang Y.L., Delaney J.C., Essigmann J.M., Greenberg M.M. (2007). Studies on the replication of the ring opened formamidopyrimidine, Fapy.dG in Escherichia coli. Biochemistry.

[18] Greenberg M.M. (2012). The formamidopyrimidines: purine lesions formed in competition with 8-oxopurines from oxidative stress. Acc. Chem. Res..

[19] Stanio S., Bacurio J.H.T., Yang H., Greenberg M.M., Basu A.K. (2023). 8-Oxo-2′-deoxyguanosine replication in mutational hot spot sequences of the p53 gene in human cells is less mutagenic than that of the corresponding formamidopyrimidine. Chem. Res. Toxicol..

[20] Bacurio J.H.T., Yang H., Naldiga S., Powell B.V., Ryan B.J., Freudenthal B.D. (2021). Sequence context effects of replication of Fapy•dG in three mutational hot spot sequences of the p53 gene in human cells. DNA Repair.

[21] Reed A.J., Suo Z. (2017). Time-dependent extension from an 8-oxoguanine lesion by human DNA polymerase beta. J. Am. Chem. Soc..

[22] Chen Y.H., Bogenhagen D.F. (1993). Effects of DNA lesions on transcription elongation by T7 RNA polymerase. J. Biol. Chem..

[23] Tornaletti S., Maeda L.S., Kolodner R.D., Hanawalt P.C. (2004). Effect of 8-oxoguanine on transcription elongation by T7 RNA polymerase and mammalian RNA polymerase II. DNA Repair.

[24] Hatahet Z., Purmal A.A., Wallace S.S. (1994). Oxidative DNA lesions as blocks to in vitro transcription by phage T7 RNA polymerase. Ann. N. Y. Acad. Sci..

[25] Brégeon D., Doddridge Z.A., You H.J., Weiss B., Doetsch P.W. (2003). Transcriptional mutagenesis induced by uracil and 8-oxoguanine in Escherichia coli. Mol. Cell.

[26] Saxowsky T.T., Meadows K.L., Klungland A., Doetsch P.W. (2008). 8-Oxoguanine-mediated transcriptional mutagenesis causes Ras activation in mammalian cells. Proc. Natl. Acad. Sci. USA.

[27] Brégeon D., Peignon P.A., Sarasin A. (2009). Transcriptional mutagenesis induced by 8-oxoguanine in mammalian cells. PLoS Genet..

[28] Oh J., Fleming Aaron M., Xu J., Chong J., Burrows Cynthia J., Wang D. (2020). RNA polymerase II stalls on oxidative DNA damage via a torsion-latch mechanism involving lone pair–π and CH–π interactions. Proc. Natl. Acad. Sci. USA.

[29] Konovalov K.A., Pardo-Avila F., Tse C.K.M., Oh J., Wang D., Huang X. (2019). 8-Oxo-guanine DNA damage induces transcription errors by escaping two distinct fidelity control checkpoints of RNA polymerase II. J. Biol. Chem..

[30] Kuraoka I., Endou M., Yamaguchi Y., Wada T., Handa H., Tanaka K. (2003). Effects of endogenous DNA base lesions on transcription elongation by mammalian RNA polymerase II: IMPLICATIONS for transcription-coupled DNA repair and transcriptional mutagenesis∗. J. Biol. Chem..

[31] Gao S., Hou P., Oh J., Wang D., Greenberg M.M. (2024). Molecular mechanism of RNA polymerase II transcriptional mutagenesis by the epimerizable DNA lesion, Fapy·dG. J. Am. Chem. Soc..

[32] Gao S., Tahara Y., Kool E.T., Greenberg M.M. (2024). Promoter dependent RNA polymerase II bypass of the epimerizable DNA lesion, Fapy•dG and 8-Oxo-2'-deoxyguanosine. Nucleic Acids Res..

[33] McAuley-Hecht K.E., Leonard G.A., Gibson N.J., Thomson J.B., Watson W.P., Hunter W.N. (1994). Crystal structure of a DNA duplex containing 8-Hydroxydeoxyguanine-adenine base pairs. Biochemistry.

[34] Ryan B.J., Yang H., Bacurio J.H.T., Smith M.R., Basu A.K., Greenberg M.M. (2022). Structural dynamics of a common mutagenic oxidative DNA lesion in duplex DNA and during DNA Replication. J. Am. Chem. Soc..

[35] You C., Dai X., Yuan B., Wang J., Wang J., Brooks P.J. (2012). A quantitative assay for assessing the effects of DNA lesions on transcription. Nat. Chem. Biol..

[36] Yang H., Tang J.A., Greenberg M.M. (2020). Synthesis of oligonucleotides containing the N6-(2-Deoxy-α,β-D-erythropentofuranosyl)-2,6-diamino-4-hydroxy-5-formamidopyrimidine (Fapy•dG) oxidative damage product derived from 2'-deoxyguanosine. Chem. - Eur. J..

[37] Greenberg M.M., Hantosi Z., Wiederholt C.J., Rithner C.D. (2001). Studies on N4-(2-Deoxy-D-pentofuranosyl)-4,6-diamino-5-formamidopyrimidine (Fapy•dA) and N6-(2-Deoxy-D-pentofuranosyl)- 6-diamino-5-formamido-4-hydroxypyrimidine (Fapy•dG). Biochemistry.

[38] Berger M., Cadet J. (1985). Isolation and characterization of the radiation-induced degradation products of 2'-deoxyguanosine in oxygen-free solutions. Z. Naturforsch..

[39] Delaney J.C., Essigmann J.M. (1999). Context-dependent mutagenesis by DNA lesions. Chem. Biol..

[40] Pande P., Haraguchi K., Jiang Y.-L., Greenberg M.M., Basu A.K. (2015). Unlike catalyzing error-free bypass of 8-OxodGuo, DNA polymerase λ is responsible for a significant part of Fapy·dG-induced G → T mutations in human cells. Biochemistry.

[41] Wang D., Bushnell D.A., Westover K.D., Kaplan C.D., Kornberg R.D. (2006). Structural basis of transcription: role of the trigger loop in substrate specificity and catalysis. Cell.

[42] Vassylyev D.G., Vassylyeva M.N., Zhang J., Palangat M., Artsimovitch I., Landick R. (2007). Structural basis for substrate loading in bacterial RNA polymerase. Nature.

[43] Damsma G.E., Cramer P. (2009). Molecular basis of transcriptional mutagenesis at 8-oxoguanine∗. J. Biol. Chem..

[44] Moriya M. (1993). Single-stranded shuttle phagemid for mutagenesis studies in mammalian cells: 8-oxoguanine in DNA induces targeted G.C-->T.A transversions in simian kidney cells. Proc. Nat. Acad. Sci. USA.

[45] Oh J., Kimoto M., Xu H., Chong J., Hirao I., Wang D. (2023). Structural basis of transcription recognition of a hydrophobic unnatural base pair by T7 RNA polymerase. Nat. Comm..

[46] You C., Wang Y. (2015). Quantitative measurement of transcriptional inhibition and mutagenesis induced by site-specifically incorporated DNA lesions in vitro and in vivo. Nat. Protoc..

