# Supplementary material for: T7 RNA polymerase catalyzed transcription of the epimerizable DNA lesion, Fapy•dG and 8-oxo-2′-deoxyguanosine
Source: J Biol Chem. 2024 Aug 29;300(9):107719. doi: 10.1016/j.jbc.2024.107719 (PMC11447338; doi:10.1016/j.jbc.2024.107719)
Supplement: Supporting Figures [file mmc1.pdf]

Supporting Information: T7 RNA Polymerase Catalyzed Transcription of the Epimerizable DNA Lesion, Fapy•dG and 8-Oxo-2'-deoxyguanosine.

Shijun Gao[a], Peini Hou[b], Dong Wang*[b,c,d] and Marc M. Greenberg*[a]

[a]Department of Chemistry, Johns Hopkins University, Baltimore, MD, 21218, USA

[b]Division of Pharmaceutical Sciences, Skaggs School of Pharmacy and Pharmaceutical Sciences, University of California, San Diego, La Jolla, CA 92093, USA

[c]Department of Cellular and Molecular Medicine, School of Medicine, University of California, San Diego, La Jolla, CA 92093, USA

[d]Department of Chemistry and Biochemistry, University of California, San Diego, La Jolla, CA 92093, USA

*Corresponding authors: dongwang@ucsd.edu, mgreenberg@jhu.edu

ORCIDs

DW: https://orcid.org/0000-0002-2829-1546

MMG: https://orcid.org/0000-0002-5786-6118

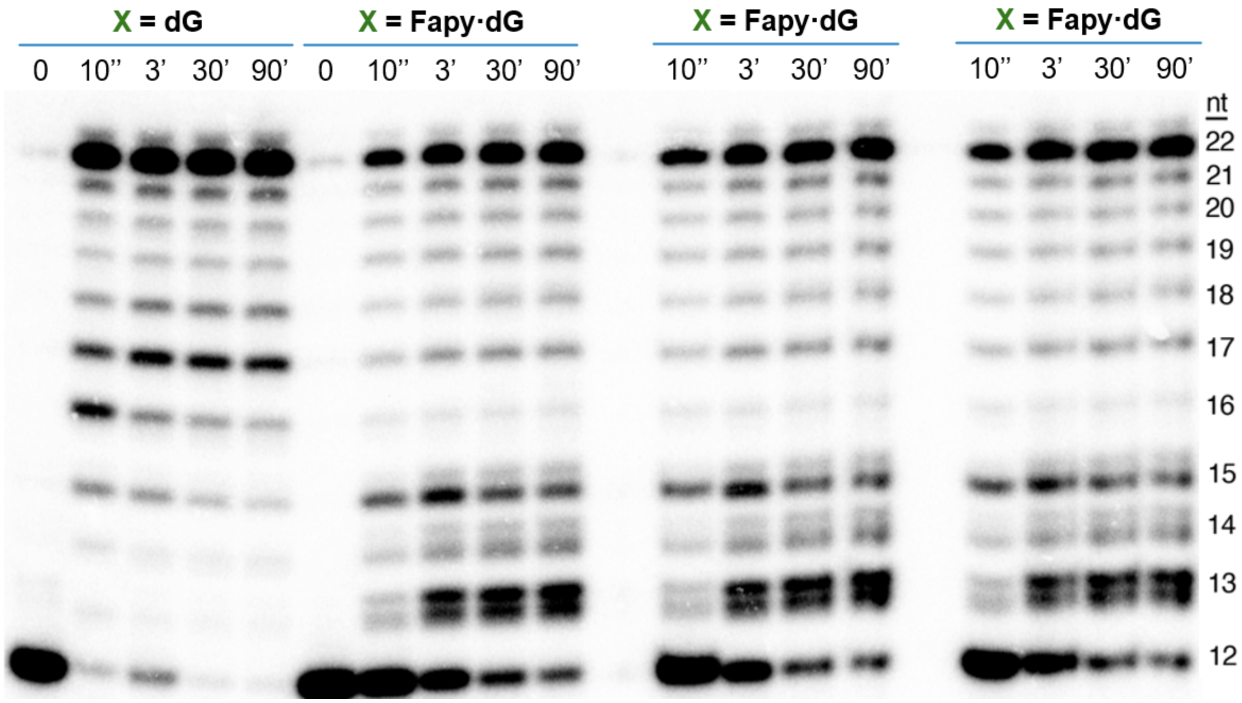

Figure S1. Additional replicates of run-off transcription using miniscaffolds **2a** and **2b**.

5'-A ACU GCG GCG AU
3'-CGC CGC TAX AGC TAA GGG
TCG ATT CCC

2b

T7 RNAP, rNTP → k_{tls}

5'-A ACU GCG GCG AUN
3'-CGC CGC TAX AGC TAA GGG
TCG ATT CCC

X = Fapy•dG; Red = RNA primer;
Black = DNA template; Blue = DNA complement

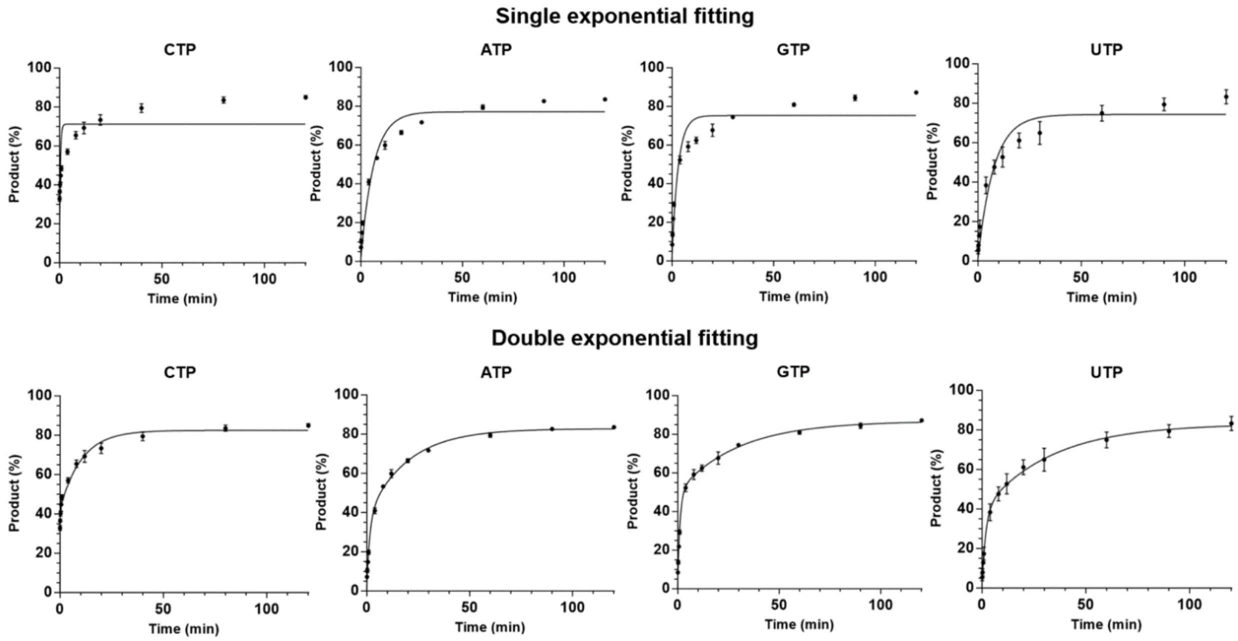

Percent Fast	51.0 ± 1.4	44.8 ± 2.5	57.4 ± 1.3	46.8 ± 3.5
k_{tls}(fast) (min^{-1})	15.5 ± 1.9	0.67 ± 0.06	0.85 ± 0.04	0.54 ± 0.08
k_{tls}(slow) (min^{-1})	9.65 ± 0.92 x 10^{-2}	5.13 ± 0.44 x 10^{-2}	3.41 ± 0.33 x 10^{-2}	3.06 ± 0.61 x 10^{-2}
% Prod. Plateau	82.4 ± 1.0	82.7 ± 0.7	86.7 ± 0.9	83.0 ± 2.1
Single exponential k_{obs} (min^{-1})	2.34 ± 0.41	0.160 ± 0.014	0.329 ± 0.045	0.130 ± 0.014

Figure S2. Single nucleotide incorporation of Fapy·dG-containing miniscaffold **2b** by T7 RNA polymerase fit to single or double exponential. Rate constants are the average ± std. dev. of 3 replicates.

5'-A ACU GCG GCG AUN
3'-CGC CGC TA**X** AGC TAA GGG
CG ATT CCC

3a-d

T7 RNAP, UTP → k_{ext}

5'-A ACU GCG GCG AUN U
3'-CGC CGC TA**X** AGC TAA GGG
CG ATT CCC

X = Fapy•dG; Red = RNA primer;
Black = DNA template; Blue = DNA complement

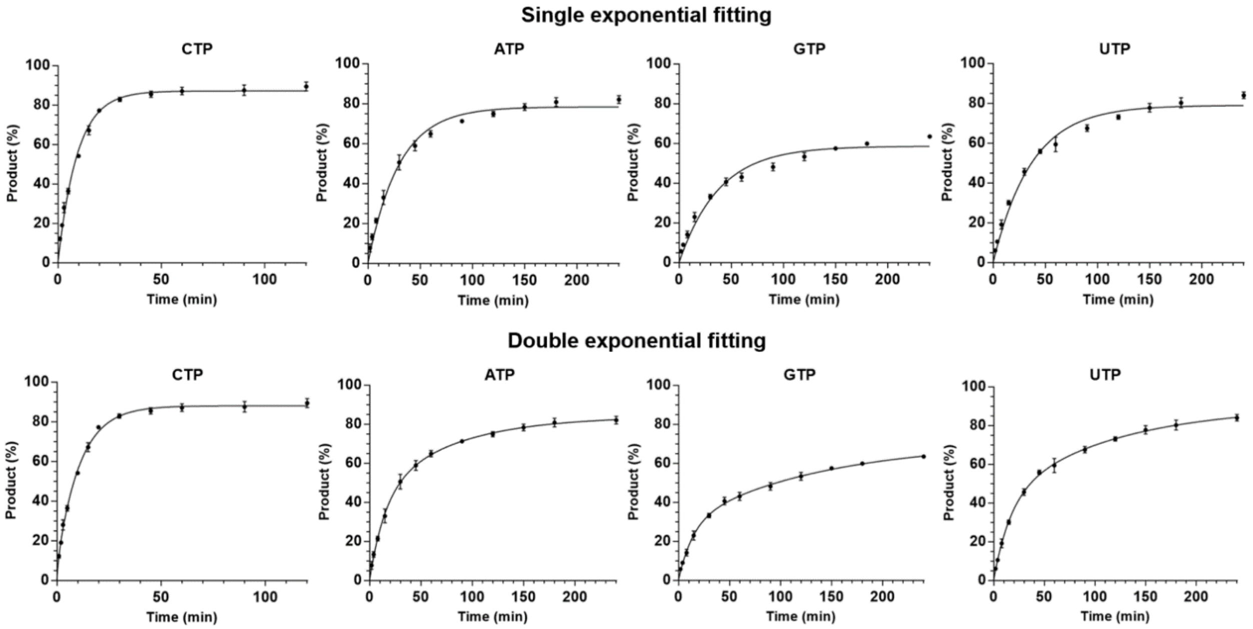

	CTP	ATP	GTP	UTP
Percent Fast	8.0 ± 2.3	50.1 ± 9.4	40.2 ± 3.0	45.4 ± 4.9
k_{ext}(fast) (min^{-1})	1.25 ± 0.84	6.68 ± 1.14 x 10^{-2}	7.17 ± 0.95 x 10^{-2}	6.06 ± 0.80 x 10^{-2}
k_{ext}(slow) (min^{-1})	0.0915 ± 0.0039	1.33 ± 0.37 x 10^{-2}	7.40 ± 1.86 x 10^{-3}	0.90 ± 0.22 x 10^{-2}
% Prod. Plateau	87.1 ± 0.7 (single exp)	84.0 ± 2.1 (double exp)	70.9 ± 4.0 (double exp)	89.9 ± 3.5 (double exp)
Single exponential k_{obs} (min^{-1})	0.11 ± 0.03	3.35 ± 0.15 x 10^{-2}	2.62 ± 0.17 x 10^{-2}	2.72 ± 1.30 x 10^{-2}

Figure S3. Extension of Fapy·dG-containing miniscaffolds **3a-d** by T7 RNA polymerase fit to single or double exponential. Rate constants are the average ± std. dev. of 3 replicates.

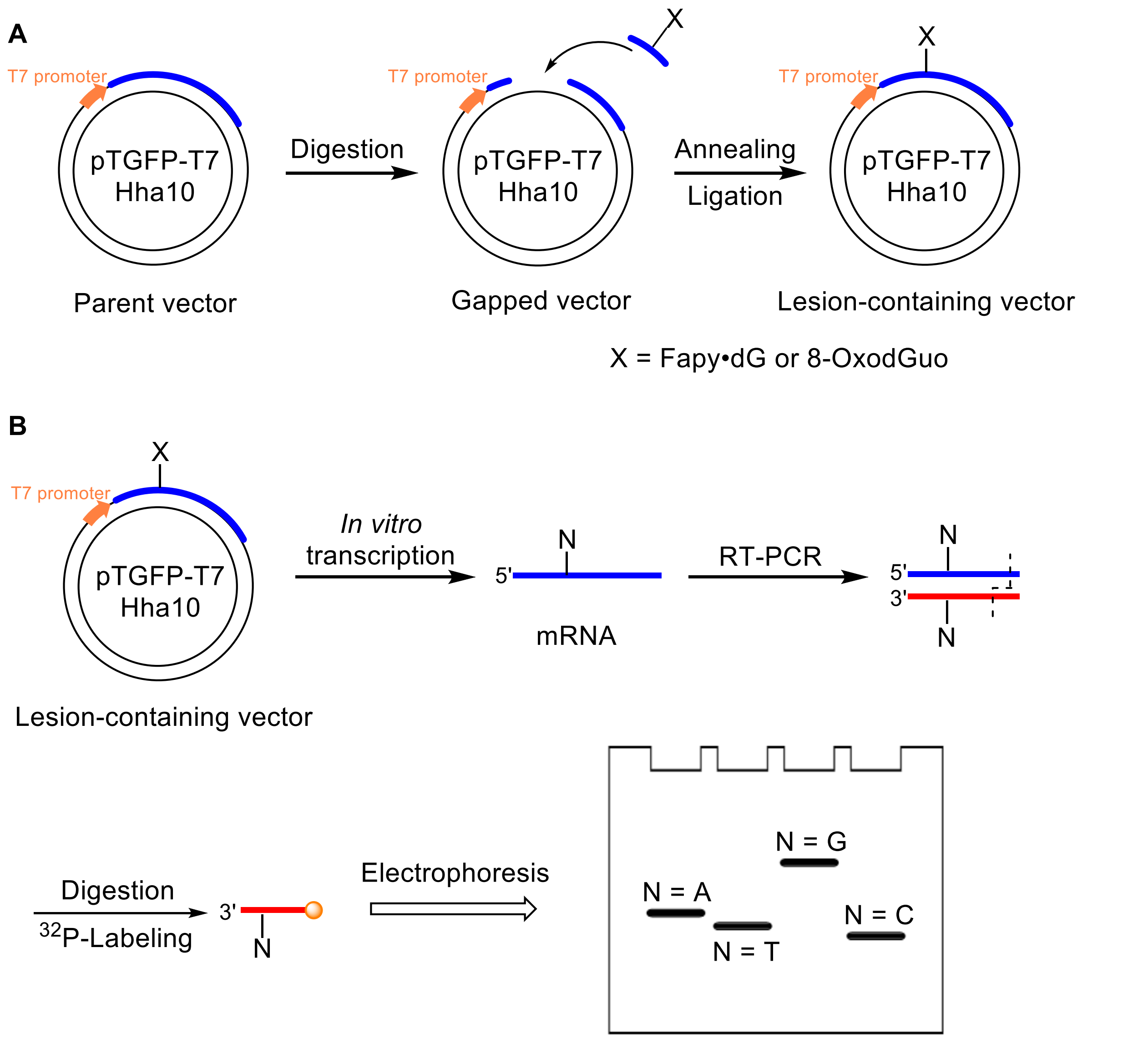

Figure S4. Overview of the CTAB method. (A) Preparation of lesion-containing vectors through a digestion-annealing-ligation process. (B) *In vitro* transcription and transcript analysis by restriction endonuclease digestion and ^{32}P-labeling.

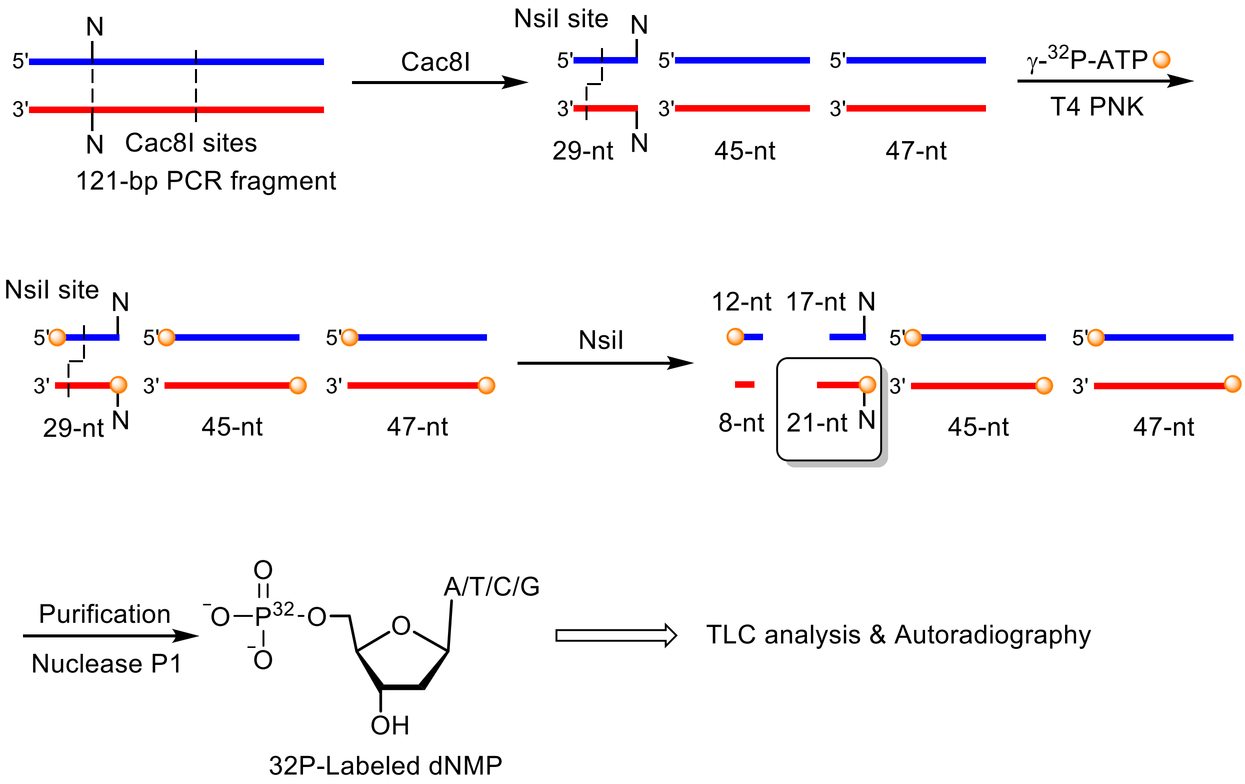

Figure S5. Processing of the 121-bp RT-PCR product for REAP assay. Digestion with Cac8I and NsiI produces four radiolabeled fragments: 12-nt, 21-nt (target), 45-nt, and 47-nt. Nuclease P1 digestion of the target fragment results in ^{32}P-labeled dNMP, which is analyzed by TLC and autoradiography.

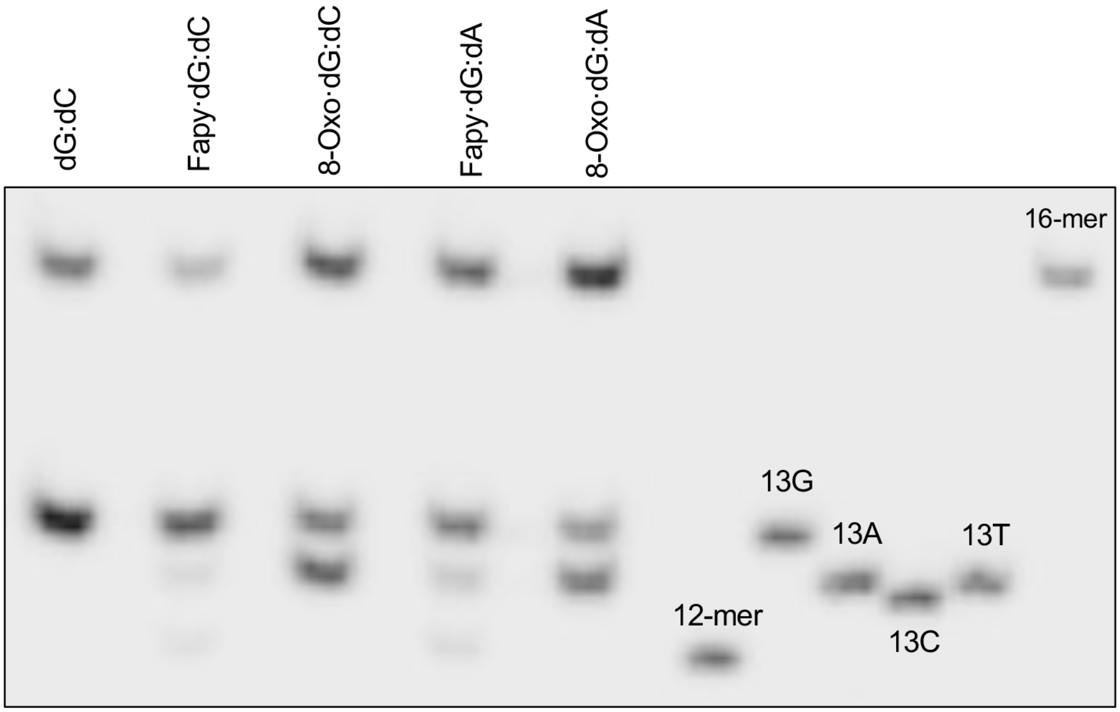

	Fapy•dG:dC	Fapy•dG:dA	8-OxodGuo:dC	8-OxodGuo:dA
**Point mutation%**	9.1 ± 0.5 %	55.1 ± 1.3 %	20.5 ± 0.1 %	55.2 ± 0.1 %
**Deletion%**	0	0	9.2 ± 0.7 %	0
**Bypass%**	149.7 ± 4.4 %	71.9 ± 5.0 %	57.4 ± 2.7%	57.5 ± 2.4 %

Figure S6. Representative gel showing the 13-mer and 16-mer fragments resulting from restriction digestion and radiolabeling during CTAB assay. The bypass % is determined using the amount of product obtained from the dG:dC standard (16-mer product) as an internal reference. Data are the ave. ± std. dev. of 3 replicates. n.d. = not detected.

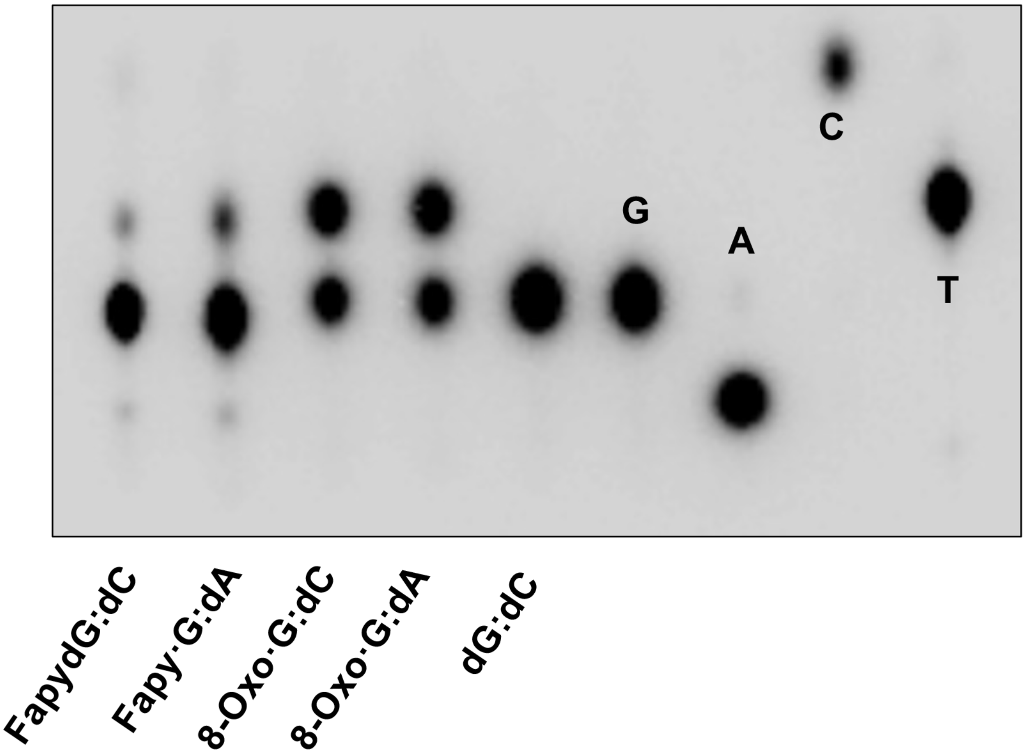

	G to T	G to A	G to C
Fapy·dG:dC	9.0±1.5%	3.0±0.3%	2.1±0.3%
Fapy·dG:dA	21.5±3.0%	4.4±1.5%	1.2±0.2%
8-OxodG:dC	61.6±2.4%	<0.1%	<0.1%
8-OxodG:dA	58.4±0.5%	<0.1%	<0.1%

Figure S7. Mutation frequency and representative TLC image for REAP analysis. G to N mutation indicates the mutation that occurs at the lesion site after the amplification of mRNA. Data are the ave. ± std. dev. of 3 replicates. n.d. = not detected.

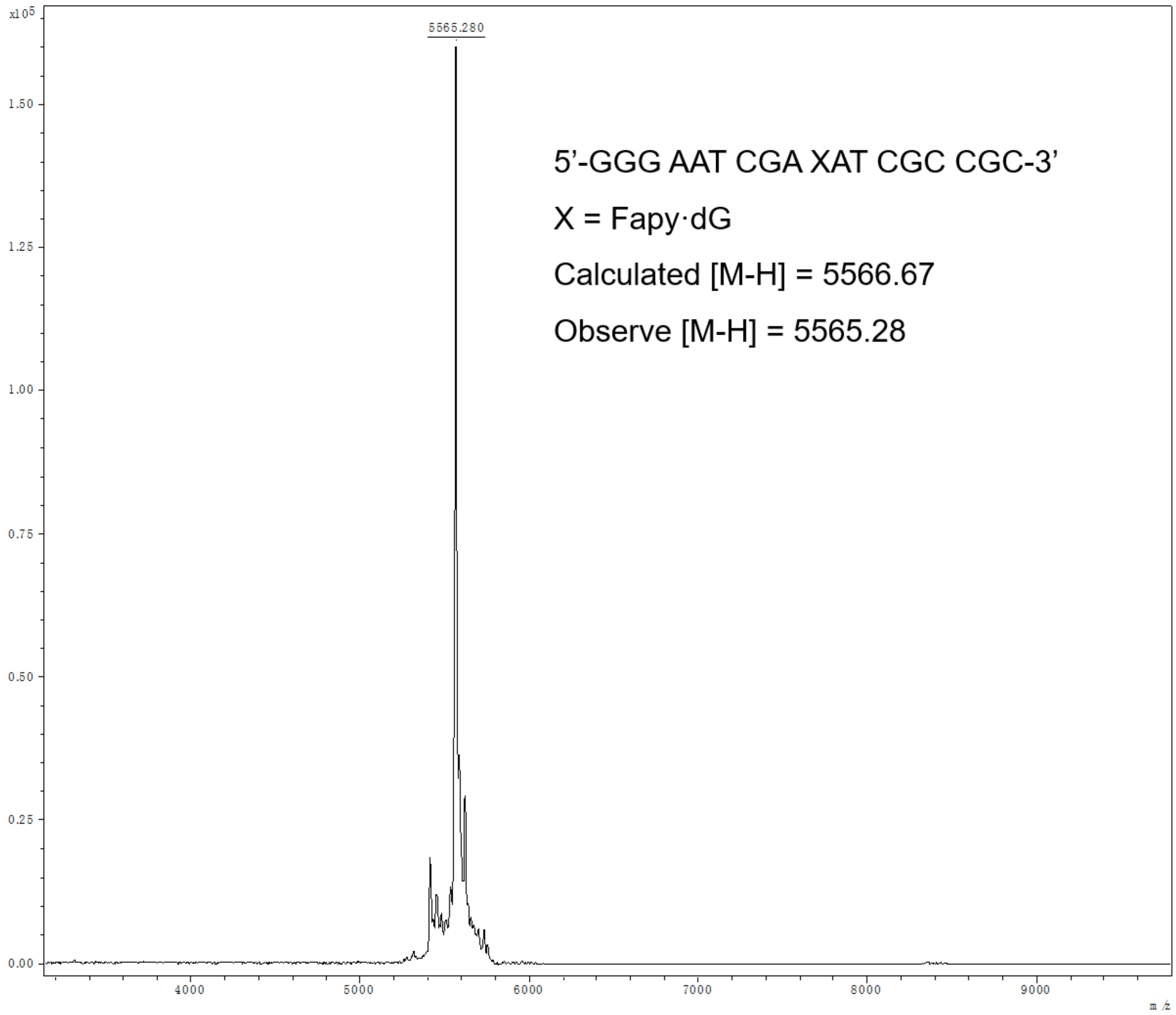

Figure S8. MALDI-TOF MS of Fapy·dG-containing DNA template used in miniscaffolds.

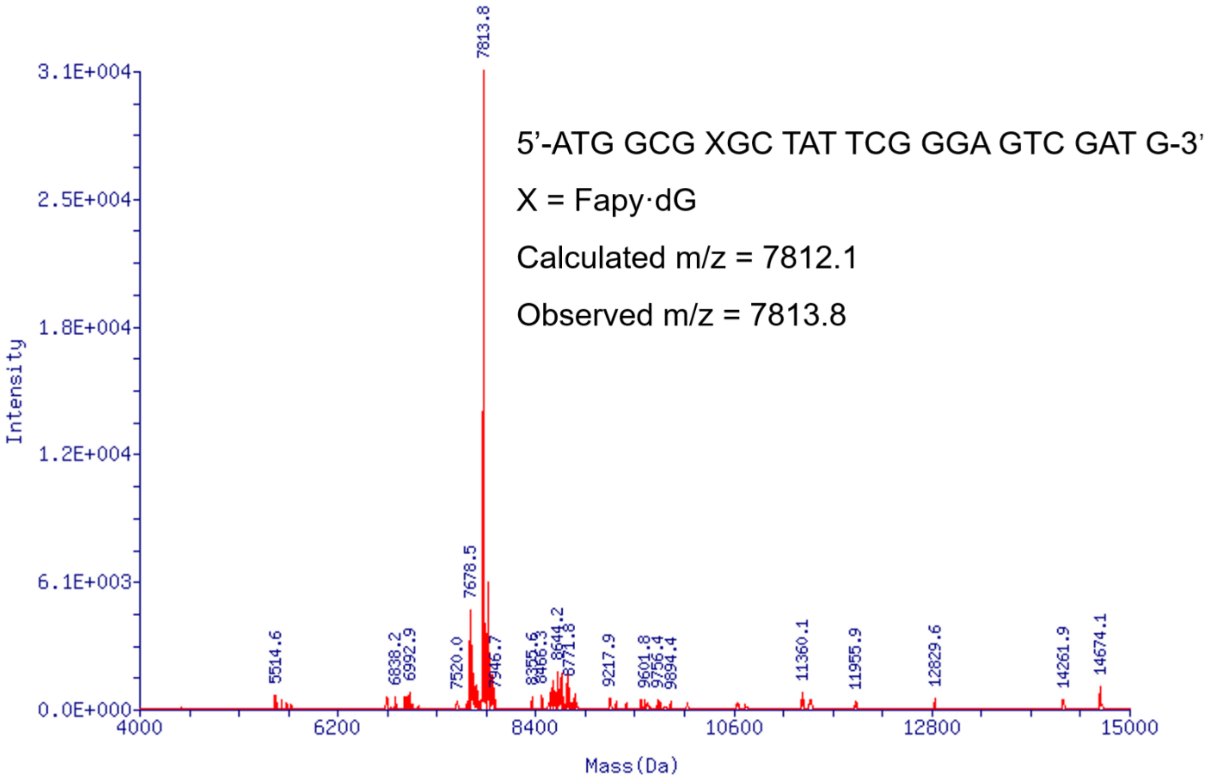

Figure S9. ESI MS of Fapy·dG-containing 25mer oligonucleotide.

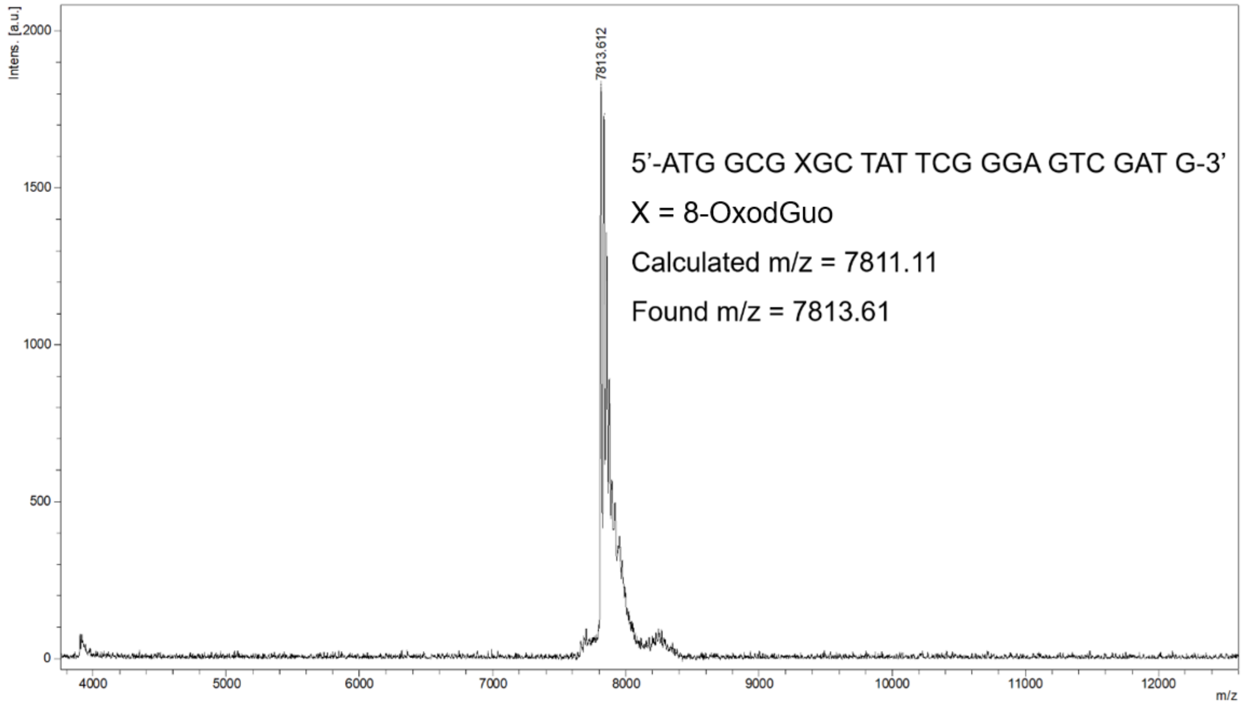

Figure S10. MALDI-TOF MS of 8-OxodGuo containing 25mer oligonucleotide.
